# The perception and knowledge of cardiovascular risk factors among medical students

**DOI:** 10.3325/cmj.2012.53.278

**Published:** 2012-06

**Authors:** Željko Reiner, Zdenko Sonicki, Eugenia Tedeschi-Reiner

**Affiliations:** 1 Department of Internal Medicine, University Hospital Centre Zagreb, University of Zagreb School of Medicine, Zagreb, Croatia; 2 Andrija Štampar School of Public Health, University of Zagreb School of Medicine, Zagreb, Croatia; 3 Sestre Milosrdnice University Hospital Center, Zagreb, Croatia; 4 University of Osijek School of Medicine, Osijek, Croatia

## Abstract

**Aim:**

To assess perceptions, knowledge, and awareness of cardiovascular disease (CVD) risk factors among medical students (freshmen and graduating students).

**Methods:**

A descriptive cross-sectional survey based on an anonymous self-administered questionnaire was conducted in 2008 on 443 medical students – 228 freshmen on their enrollment day and 214 students on the day of their final exam at the University of Zagreb School of Medicine, Croatia.

**Results:**

The perception and knowledge of some CVD risk factors, eg, dyslipidemia, arterial hypertension, and metabolic syndrome as well as of lipid-lowering therapy important for CVD prevention was significantly better among graduating students but was still not sufficient. Only 66% of graduating students reported that they would prescribe lipid-lowering therapy to high risk patients. Disappointingly, many graduating students were smoking (30.4%) and had low-awareness of obesity as an important CVD risk factor.

**Conclusion:**

These results suggest an urgent need to improve medical students’ knowledge of obesity and low physical activity as important CVD risk factors and of the methods for increasing low high-density lipoprotein-cholesterol and for smoking cessation. All this provides a rationale for modifying the university core curriculum to include more information concerning these issues.

Although over the past 25 years death rates of cardiovascular diseases (CVDs) have been decreasing in most northern and western European countries and the USA, they have been increasing in most central and eastern European countries and are still the number one cause of death in Europe, responsible for 48% of all deaths (1).

CVD has multiple risk factors, the most important being dyslipidemias, high blood pressure (BP), smoking, overweight and obesity, low physical activity, diabetes, and metabolic syndrome (2). Almost all of them are modifiable, suggesting that most of CVDs are preventable (3).

Perceptions, knowledge, and awareness of CVD risk factors among medical students have not yet been studied and there are no data on how they change during medical education or how successful medical education is in increasing the awareness of the need for prevention in cardiology. Since knowledge and perception of these risk factors is very important for medical students and young physicians, the aim of this PERCRO-STUD (PERception of cardiovascular risk factors by CROatian medical STUDents) cross-sectional survey was to assess the perceptions, knowledge, and awareness of CVD risk factors in medical students.

## Methods

An anonymous self-administered questionnaire with 25 multiple choice questions composed for the purposes of this survey (web-extra material) (web extra material) was answered by all 228 students entering the University of Zagreb School of Medicine on their enrollment day in 2008, as well as by 214 students on the day of their final graduation exam in July and September 2008. This second group consisted of all the students who took their final graduation exam. So, two different generations were interviewed, one at the beginning and one at the end of their medical education. The questionnaire involved numerical information (eg, **“**What is the recommended level for total plasma cholesterol in mmol/L in subjects without coronary heart disease?” or “What is the recommended blood pressure for subjects with high risk for cardiovascular diseases in mmHg?”) or conversion of non-numerical data to a numerical format by the use of ranking scales (eg, “Rank on a scale of 1 to 10 cardiovascular risk factors listed bellow according to their relevance beginning with number 1 for the most relevant to number 10 for the least relevant.”). A very similar questionnaire containing 80% of the same questions was validated on general population of Croatia (4). The study and the questionnaire were approved by the Ethics Committee of the University of Zagreb School of Medicine.

Collected data were described by frequencies and percentages. Ranking order of risk factors, obtained by the use of rating scales, was assessed by median values and for equal medians by mode values. Chi-square statistics, or two-sided Fisher exact test when appropriate, were used to asses the association of certain questionnaire items. Data analysis was performed by statistical software R, version 2.10.1 (5).

## Results

The proportion of women among 228 freshmen was 69.3% and among 214 graduating students it was 65.3%. A total of 71.9% freshmen knew their blood pressure (BP) but only 13.6% knew their total plasma cholesterol (TC). Of graduating students, 91.6% knew their BP (χ^2^_1_ = 28.234; *P* < 0.001) and 40.2% their TC (χ^2^_1_ = 40.101; *P* < 0.001). Only 14% of freshmen were smokers and 0.9% were past smokers, while 30.4% of graduating students were smokers and 4.7% were past smokers (χ^2^_2_ = 25.525; *P* < 0.001) (Table 1).

**Table 1:** Medical students’ answers at the beginning and at the end of their education

	Beginning of education		End of education		
	**N**	**%**	**N**	**%**	***P***
Most feared disease:					
cancer	139	61.0	143	66.8	<0.001*
cardiovascular diseases	31	13.6	46	21.5	
AIDS	44	19.3	16	7.5	
liver diseases	7	3.1	3	1.4	
lung diseases	4	1.8	1	0.5	
no answer	3	1.3	5	2.3	
total	228	100.0	214	100.0	
Leading cause of death is:					
traffic accidents	39	17.1	9	4.2	<0.001*
cancer	39	17.1	8	3.7	
cardiovascular diseases	148	64.9	196	91.6	
AIDS	0	0.0	1	0.5	
liver diseases	1	0.4	0	0.0	
no answer	1	0.4	0	0.0	
total	228	100.0	214	100.0	
Smoking habits:					
smokers	32	14.0	65	30.4	<0.001^†^
non-smokers	194	85.1	138	64.5	
past smokers	2	0.9	10	4.7	
no answer	0	0.0	1	0.5	
total	228	100.0	214	100.0	
Knowledge of Joint European Guidelines on CVD Prevention (2):					
very good	8	3.5	18	8.4	<0.001^†^
partial	51	22.4	126	58.9	
no knowledge at all	95	41.7	29	13.6	
just heard about it	73	32.0	39	18.2	
no answer	1	0.4	2	0.9	
total	228	100.0	214	100.0	
Guidelines (2) goal value for total cholesterol in apparently healthy subjects:					
<5 mmol/L	83	36.4	117	54.7	<0.001*
<5.2 mmol/L	91	39.9	79	36.9	
<6.5 mmol/L	34	14.9	9	4.2	
<7.8 mmol/L	4	1.8	2	0.9	
no answer	16	7.0	7	3.3	
total	228	100.0	214	100.0	
Guidelines-recommended value for high-density lipoprotein-cholesterol in women (2):					
<0.9 mmol/L	11	4.8	4	1.9	<0.001^†^
>0.9 mmol/L	25	11.0	33	15.4	
>1 mmol/L	34	14.9	55	25.7	
<1 mmol/L	49	21.5	5	2.3	
>1.2 mmol/l	34	14.9	105	49.1	
<1.2 mmol/L	42	18.4	3	1.4	
no answer	33	14.5	9	4.2	
total	228	100.0	214	100.0	
Guidelines-recommended blood pressure for high-risk subjects (2):					
<150/90 mm Hg	22	9.6	4	1.9	<0.001^†^
<140/90 mm Hg	26	11.4	41	19.2	
<135/85 mm Hg	65	28.5	94	43.9	
<120/80 mm Hg	105	46.1	70	32.7	
no answer	10	4.4	5	2.3	
total	228	100.0	214	100.0	
Would you prescribe lipid lowering therapy to high risk subjects:					
yes	21	9.2	138	64.5	<0.001^†^
no	28	12.3	56	26.2	
do not know	161	70.6	15	7.0	
no answer	18	7.9	5	2.3	
total	228	100.0	214	100.0	
Metabolic syndrome:					
causes directly diabetes	7	3.1	13	6.1	<0.001^†^
significantly increases coronary risk	37	16.2	161	75.2	
is a rare metabolic disease	16	7.0	9	4.2	
has low coronary risk. Causes some other diseases	24	10.5	15	7.0	
do not know anything about	118	51.8	4	1.9	
no answer	26	11.4	12	5.6	
total	228	100.0	214	100.0	
Possibilities for raising HDL-cholesterol:					
no carbohydrate and no alcohol intake	26	11.4	18	8.4	<0.001^†^
moderate alcohol intake and regular physical activity	19	8.3	76	35.5	
low saturated animal fat diet	116	50.9	102	47.7	
apple vinegar	20	8.8	5	2.3	
ginco biloba	13	5.7	5	2.3	
no answer	34	14.9	8	3.7	
total	228	100.0	214	100.0	
Opinion on combined lipid-lowering therapy (life-style +2 or more drugs):					
must not be given because too high risk of side-effects	9	3.9	7	3.3	<0.001^†^
appropriate for high hypercholesterolemia and high hypertriglyceridemia	18	7.9	132	61.7	
only for high hypercholesterolemia and low HDL cholesterol	32	14.0	29	13.6	
do not know	146	64.0	36	16.8	
no answer	23	10.1	10	4.7	
total	228	100.0	214	100.0	

*Two-sided Fisher exact test.

†χ^2^ test.

Both groups of students perceived cancer as the most feared disease (Table 1). However, freshmen ranked AIDS second and CVD third and graduating students ranked CVD second and AIDS was significantly much less feared (*P* < 0.001, two-sided Fisher exact test).

Freshmen perceived CVD as the leading cause of death and cancer and traffic accidents as the second. Graduating students significantly more often perceived CVD as the leading cause of death and significantly less often cancer and traffic accidents (*P* < 0.001, two-sided Fisher exact test).

Significantly more graduating students than freshmen reported either good or partial knowledge of the last Joint European Guidelines on CVD Prevention (χ^2^_3_ = 80.658, *P* < 0.001). Still, 45.3% of graduating students did not answer correctly on the question about the Joint European Guidelines goal value for TC for clinically healthy persons. The same was true for the question on high density lipoprotein (HDL)-cholesterol, to which only half of the graduate students answered correctly (χ^2^_5_ = 115.065; *P* < 0.001), as well as for the question on BP, where also only half of the students knew the target values for high-risk subjects (χ^2^_3_ = 27.932; *P* < 0.001). Most of graduate students reported that they would prescribe lipid-lowering therapy to all the high-risk patients (66%), which is much more than among freshmen, only 10% of whom would prescribe such a treatment (χ^2^_2_ = 216.540, *P* < 0.001).

**Figure 1:**
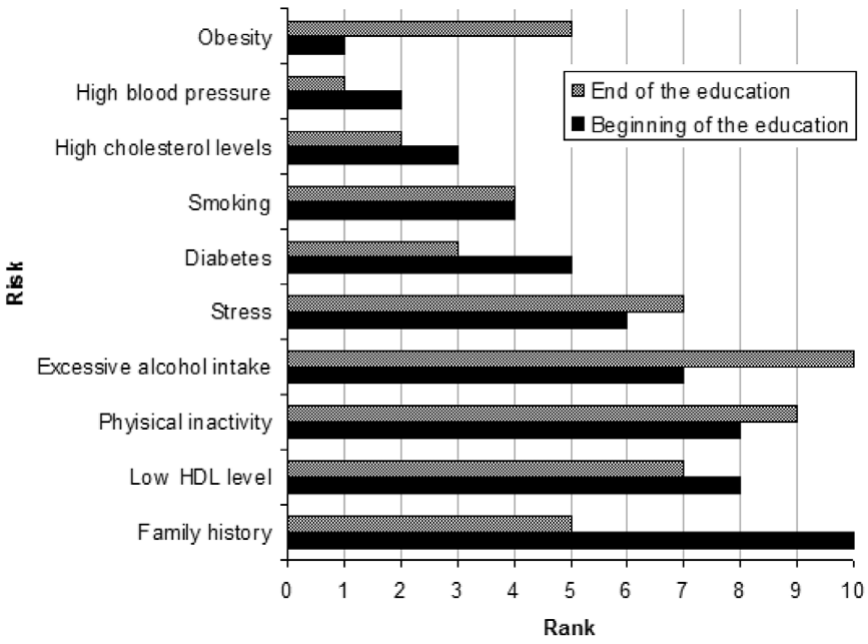
Knowledge about the risk rank among freshmen (closed bars) and graduates (gray bars) (the smaller the bars the higher the rank).

When asked to rank the factors that increase the CVD risk, graduating students least commonly reported excessive alcohol intake and physical inactivity but ranked obesity much lower than freshmen (Figure 1). Smoking was ranked equally by both groups.

The knowledge on metabolic syndrome was significantly better among graduating students than among freshmen (χ^2^_4_ = 190.018; *P* < 0.001). However, although the knowledge of the possibilities for raising the low HDL-cholesterol was significantly better among graduating students than among freshmen (χ^2^_4_ = 48.793; *P* < 0.001), still half of the graduating students reported that they would advise their patients only to decrease dietary saturated fat of animal origin to achieve this.

Much more graduating students than freshmen reported that they would prescribe combined lipid-lowering therapy (χ^2^_3_ = 153.520; *P* < 0.001). A total of 28.5% of graduating students believed that they had not learned enough about risk factors for CVD.

## Discussion

This study showed that perception and knowledge of some CVD risk factors was significantly better among graduating students than among freshmen but was still not sufficient. Freshmen’s knowledge and attitudes did not differ much from those of other young people of their age (6). It was better than, for example, the knowledge of students of Michigan high schools, who although rated accidents as the greatest perceived lifetime health risk, identified CVD as the greatest cause of death (6). However, it was about the same as the Croatian general population’s knowledge about causes of death (4).

Although the knowledge on CVD risk factors in our students, as expected, was significantly better at the end of university medical education, the results of this survey suggest insufficient awareness of CVD risk factors and indicate an urgent need for an improved promotion of CVD prevention during medical education. This might be a problem of the curriculum, which comprises mandatory courses in family medicine, epidemiology, and public medicine as well as internal medicine, during which students have only about 4 hours of lectures, 6 hours of seminars, and 12 hours of practicals and clinical audits on CVD risk factors and prevention. However, they discuss CVD risk factors in a number of other clinical audits. Despite the fact that most of graduating students believed they were familiar with last Joint European guidelines on CVD prevention (2), too many of them did not know the target values for TC, HDL-cholesterol, and BP, and only 64.5% reported that they would prescribe lipid-lowering therapy to high-risk subjects. Therefore, if medical education is like this, it is not surprising that several recent studies have shown a failure to achieve the recommended risk factor targets in patients with CVD and those without CVD but with CVD risk factors not only in Croatia but in many other European countries (7-12).

A very disturbing fact is that many students were smoking at the end of their medical education in spite of sufficient knowledge about harmful effects of smoking (14% vs 30.4%). This is in accordance with the data from two Spanish surveys. One of them showed that 27% of final year medical students were smokers and 32.54% of them had started smoking during their medical studies (13). The other showed that the prevalence of smokers among Spanish medical students increased between the first study year and the beginning of the third year from 20% to 31% (14). A survey performed in 2010 on students of four Italian medical schools showed that they had limited knowledge about tobacco dependence, how to treat it, and the critical role of the physician in promoting smoking cessation (15).

Similarly disappointing is graduating students’ low awareness of obesity as an important CVD risk factor. Namely, recent data show that the prevalence of obesity is increasing and reaching epidemic proportions, particularly in the high-risk group of patients with CVD all over Europe and that management of excessive body weight, which is at the moment inadequate, should be given the highest priority (16).

It is encouraging that graduating students had relatively good knowledge on metabolic syndrome and atherogenic dyslipidaemia characterized by low HDL-cholesterol and elevated triglycerides. These are typically encountered in high-risk patients with metabolic disorders like diabetes and/or obesity, which have an increasing prevalence but are largely under-diagnosed and under-treated (17-19). In fact, their knowledge of HDL-cholesterol was not much worse than the knowledge of Croatian general practitioners and/or family doctors, although this was also not satisfactory (20). However, students’ knowledge on increasing low HDL-cholesterol was clearly not sufficient. Another encouraging finding is graduating students’ quite positive attitude toward combined lipid-lowering treatment consisting of two or more different lipid-lowering drugs, which was feared by many physicians until very recently, mainly because of adverse effects (21).

The major limitation of the study is that it compared two different generations of students, so no clear conclusion about the success of medical education can be made. For this purpose, the same population of medical students should be evaluated at the beginning and at the end of their education.

Based on the results presented, it could be concluded that university medical education on CVD prevention, at least in Croatia, must be substantially improved and should include strategies to increase not only knowledge but also perception of modifiable risk factors for CVD and strategies to reduce or eliminate them. Particular attention has to be paid to increase students’ knowledge about obesity and low physical activity as important CVD risk factors, but also to the methods for increasing low HDL-cholesterol and smoking cessation. To achieve this, the core curriculum should be modified to include more information about these issues.
