# Supplementary material for: The perception and knowledge of cardiovascular risk factors among medical students
Source: Croat Med J. 2012 Jun;53(3):278–84. doi: 10.3325/cmj.2012.53.278 (PMC3378972; doi:10.3325/cmj.2012.53.278)
Supplement: Supplementary material 1 [file CroatMedJ_53_s002.pdf]

WHAT DO YOU KNOW ABOUT RISK FACTORS FOR CARDIOVASCULAR AND CEREBROVASCULAR DISEASES?
(unless specifically stated, you have to round letter in front of only one correct answer)

1. **You are:**

   a) student getting enrolled in School of Medicine, University of Zagreb
   b) student taking final graduation exam

2. **Your sex is:**

   a) male
   b) female

3. **Which of the following diseases listed bellow are you most affright of?**

   a) cancer or some malignant diseases
   b) stroke and similar cerebrovascular diseases
   c) AIDS (SIDA)
   d) myocardial infarction and similar heart and blood vessels diseases
   e) cirrhosis and other liver diseases
   f) tuberculosis and other lung diseases

4. **Do you know your blood pressure?**

   a) yes
   b) no

5. **Do you know your total cholesterol plasma concentration?**

   a) yes
   b) no

6. **Do you smoke?**

   a) yes
   b) no
   c) I am a former smoker

7. **According to your knowledge, what is the leading cause of death in Croatia?**

   a) traffic accidents
   b) cancer or some malignant disease
   c) stroke and similar cerebrovascular diseases
   d) AIDS (SIDA)
   e) myocardial infarction and similar cardiovascular diseases
   f) cirrhosis and other liver diseases
   g) tuberculosis and other lung diseases

8. Which is the 'bad' atherogenic cholesterol which if elevated increases cardiovascular risk:

a) **HDL cholesterol**
b) **LDL cholesterol**
c) **triglycerides**
d) **none of these**

9.Which is "protective" antiatherogenic cholesterol which if elevated does not increase the cardiovascular risk?

a) **HDL cholesterol**
b) **LDL cholesterol**
c) **triglycerides**
d) **none of these**

10. Are you familiar with the last Joint European guidelines on cardiovascular disease prevention in clinical practice published in the year 2007?

a) **yes, I have good knowledge about them**
b) **I have only partial knowledge about them**
c) **no, I have no knowledge about them at all**
d) **I have heard about them, but I do not know them**

11. Rank (according to your knowledge) cardiovascular risk factors listed bellow according to their relevance (write a number in front of each risk factor, beginning with number 1 (one) for the most relevant, to number 10 (ten) for the least relevant):

Diabetes
Elevated total plasma cholesterol
High blood pressure
Low „protective" HDL cholesterol
Smoking
Obesity
Stress
Excessive alcohol drinking
History of myocardial infarction or stroke in family
Lack of physical activity, sport and exercise

12. According to your knowledge, what is the recommended level for total plasma cholesterol (expressed in mmol/L) in subjects <u>without</u> coronary heart disease?

a) less than 5
b) less than 5,2
c) less than 6,5
d) less than 7,8

13. According to your knowledge, what is the recommended level for HDL cholesterol in women (expressed in mmol/L)?

a) **less than 0,9**
b) **more than 0,9**
c) **more than 1,0**
d) **less than 1,0**
e) **more than 1,2**
f) **less than 1,2**

14. According to your knowledge, a patient with diabetes should have LDL cholesterol level (expressed in mmol/L):

a) **less than 5**
b) **less than 4**
c) **less than 3**
d) **less than 2,5**

15. According to your knowledge, what is the recommended blood pressure for subjects with high risk for cardiovascular diseases is (in mmHg):

a) **less than 150/90**
b) **less than 140/90**
c) **less than 130/85**
d) **less than 120/80**

16. Is it important to increase very low HDL cholesterol in patients with high risk for coronary heart disease?

a) **yes, it is very important**
b) **it is relatively important**
c) **it is not so important**
d) **it is not important at all, important are the other lipid parameters**

17. Which of the following statements about metabolic syndrome is correct?

a) **it always causes diabetes**
b) **it increases significantly cardiovascular risk**
c) **this is a rare metabolic disease**
d) **it increases cardiovascular risk only very slightly, but is important because of the potential impact on other diseases**
e) **I do not know what metabolic syndrome is**

18. Would you always prescribe lipid lowering drug to patients with a history of myocardial infarction, proven coronary artery disease, diabetes, stroke, transient ischemic attacks and/or proven carotid plaque?

a) yes
b) no
c) I do not know

19. According to your knowledge, in most patients by using only dietary measures you can reduce total cholesterol for:

a) 2 – 3%
b) 10 – 15 %
c) about 50 %
d) 50 – 75 %
e) up to 90 %

20. What would you prescribe to a patient with significantly low HDL cholesterol, moderately elevated triglycerides and normal or only slightly elevated total and LDL cholesterol?

a) nothing, because this condition should not be treated
b) nothing, because we still lack an effective drug to increase significantly HDL cholesterol
c) statin
d) fibrate
e) I do not know

21. HDL cholesterol can be significantly increased with:

a) diet with no carbohydrates and total abstinence from alcohol
b) moderate alcohol consumption and regular increased physical activity/exercise
c) diet with low saturated fat of animal origin
d) increased consumption of apple cider vinegar
e) ginko biloba

22. Which statement is the most acceptable when it comes to combined drug treatment of dyslipidaemia?

a) it should not be prescribed because the risk of adverse effects is too high
b) it should be prescribed only to those who have extreme hypercholesterolaemia and extreme hypertriglyceridaemia
c) it should be prescribed only to those with extreme hypercholesterolemia who also have very high HDL cholesterol
d) I do not know

23. Do you think you have learned enough about risk factors for cardiovascular and cerebrovascular diseases during your medical education ? (ONLY STUDENTS TAKING GRADUATE EXAM SHOULD ANSWER THIS QUESTION):

a) yes
b) no
